# Evaluation of Torula Yeast (*Candida utilis*) Single Cell Proteins as a Partial Replacement for Soybean Meal in Broiler Diets: Effects on Productive Performance, Meat Quality Traits, and Plasma Metabolome

**DOI:** 10.1111/jpn.70082

**Published:** 2026-06-01

**Authors:** Jonathan Dayan, Marco Zampiga, Francesca Soglia, Luca Laghi, Elisa Benini, Massimiliano Petracci, Federico Sirri

**Affiliations:** ^1^ Department of Agricultural and Food Sciences Alma Mater Studiorum ‐ University of Bologna Cesena Italy; ^2^ Department of Veterinary Medical Sciences Alma Mater Studiorum ‐ University of Bologna Cesena Italy

**Keywords:** alternative proteins, broiler nutrition, growth performance, meat quality, plasma metabolome

## Abstract

Over recent years the use of yeast in feedstuffs has gained momentum due to novel developments in single‐cell proteins (SCPs). SCPs are considered a sustainable protein source, as their production enable to convert low‐value substrates (including industrial/agricultural wastes) into feed, with a related reduced environmental impact. This study provides a first‐step insight into the dietary inclusion of torula yeast‐derived SCPs in broiler chicken diets. A total of 720 1‐day‐old male broilers were divided into four groups (10 replicate pens/group): the control group (CON) received a commercial soybean‐based diet, while the SCP groups (SCP2, SCP4, SCP6) were fed diets supplemented with 2%, 4%, or 6% SCPs during the grower and finisher phases (15–42 d) as a partial replacement for soybean meal. Growth performance, footpad dermatitis (FPD), plasma metabolome, and breast‐meat quality and composition were evaluated. For the entire trial period, performance traits and FPD rates did not differ significantly, however a trend was observed for body weight and daily weight gain which tended to decrease with increasing SCP levels (3083, 3058, 2988, 2942 g/bird, and 72.1, 71.6, 69.8, 68.8 g/bird/day respectively for CON, SCP2, SCP4, and SCP6; *p* = 0.071; linear fit < 0.01). Breast‐meat quality and composition were maintained apart from linearly increased yellowness (b*) in CON, SCP2, SCP4, and SCP6 (7.67, 8.57, 9.11, and 10.38; *p* < 0.001; linear fit < 0.01). As for plasma metabolites, focusing on differences attributable to SCPs inclusion levels, on day 21, SCP6 reduced Threonine (−34%) and increased Isoleucine (+26%), Ethanol (+91%), Uridine (+67%), Leucine (+24%), and 2,3‐butanediol (+60%) compared with CON (*p* < 0.05). At day 42, SCP6 reduced Arginine (−46%) and Threonine (−17%) and increased 2‐aminobutyrate (+51%), Uridine (+116%), Trans‐4‐hydroxy‐L‐proline (+29%), and Formate (+25%) (*p* ≤ 0.05). Overall, the findings demonstrate the feasibility of graded SCPs inclusion in broiler diets, with a potential upper limit of 4%, as indicated by changes in performance and plasma metabolome.

## 1 Introduction

The use of yeast as an ingredient in animal and specifically poultry feeding has long been recognized and was even regarded as a breakthrough stage at the time (Ringrose 1949; Vananuvat and Balloun 1977). Nevertheless, in more recent years, its application has accelerated and gained momentum due to novel developments in single‐cell proteins (SCPs) for feed and food (Buerth et al. 2016; Ledbetter 2025). The term SCPs was first mentioned about 60 years ago and refers to dried biomass of micro‐organisms, including bacteria, fungi, microalgae, and, of course, yeast, produced through fermentation processes (Litchfield 1983; Ugalde and Castrillo 2002). SCPs are considered a sustainable alternative protein source as microorganisms can convert low‐value substrates, including industrial and agricultural by‐products, in food and feed proteins through consistent year‐round production (Sharif et al. 2021; Albrektsen et al. 2022). In addition, their low land and water requirements, and reduced greenhouse gas emissions, further support their role as a circular, environmentally sustainable protein source to meet growing global demand (Sharif et al. 2021; Hooft et al. 2024). Identifying alternative protein sources is a priority for the poultry industry to support projected future production growth. Despite the relatively low environmental footprint of poultry compared with other livestock sectors, reliance on soybean meal represents a major sustainability bottleneck (Pope et al. 2024; Zampiga et al. 2024).

From a nutritional point of view, as recently presented by Busti et al. (2025), yeast‐derived SCPs have a relatively high protein content (up to 55%) and an overall balanced amino acid, vitamins, and minerals composition. Yeast‐derived SCPs may lack some sulphur‐containing amino acids, such as methionine and cysteine, but are rich in essential amino acids like lysine and leucine. SCPs also contain a non‐digestible cell wall, which may pose a challenge for monogastric animals and therefore requires its degradation (Ritala et al. 2017). However, the beneficial effects of cell wall components like mannan‐oligosaccharides and β‐glucans on growth performance, intestinal morphology, and immunity of broiler chickens are well documented (Cox and Dalloul 2010; Cheled‐Shoval et al. 2011, 2014; Chacher et al. 2017; Teng et al. 2021; Bar‐Dagan et al. 2023). Among yeast‐based SCPs, Torula yeast (*Candida utilis*) has been one of the most extensively studied over the past decades, with demonstrated applications primarily in monogastric animals such as fish (Busti et al. 2025) and pigs (Espinosa et al. 2020; Lagos and Stein 2020). In contrast, research evaluating the use of yeast‐based SCPs in broiler chickens remains limited. In our preliminary study, inclusion dosages of 10% and 15% up to 21 days of age had significant adverse effects on all productive performance traits (unpublished data). To further explore the potential of SCP in broiler chicken diets, a dose‐response study was carried out to evaluate the effects of moderate graded dietary dosages (from 2% to 6%) of yeast‐derived SCPs as an alternative to soybean meal on growth performance, meat quality traits, and plasma metabolomic profile of broiler chickens.

## 2 Materials and Methods

### 2.1 Birds, Husbandry and Growth Performance Traits

A total of 720 1‐day‐old Ross 308 male chicks were divided into four experimental groups and reared from the day of hatch until day 42 (10 pens/group and 18 birds/pen). The birds were housed in the poultry facility of the Department of Agricultural and Food Sciences, Alma Mater Studiorum ‐ University of Bologna, located in Ozzano dell'Emilia, Italy (Via del Florio 2, 40064). Birds were randomly allocated to concrete‐floor pens (3 m^2^ each) using wood shavings (2 kg/m2) as litter material. Stocking density followed the EU legislation in force (Dir. 2007/43/EC) with a maximum of 33 kg live weight/m^2^). Each pen was provided with a circular pan feeder able to guarantee at least 2 cm of front space/bird and an independent drinking system with 1 nipple/5 birds. The environmental temperature and humidity were defined according to the management guide provided by the breeding company. According to the EU legislation (Dir. 2007/43/EC), photoperiod was 23 h light to 1 h dark from 0 to 7 d, whereas 18 h light to 6 h dark was used from 8 d to slaughtering. Twice daily observations recorded general flock condition, temperature, lighting, water, feed, litter condition, and mortality. A three‐phase feeding programme was used (starter: 0–14 d; grower: 15–29 d; and finisher: 30–42 d). No coccidiostats were included in the feed as chicks were vaccinated at the hatchery. Feed was administered in a mash form for ad libitum consumption (water was supplied on an ad libitum basis as well). The control (CON) group received a commercial diet with soybean as the main protein source in all feeding phases. The SCP2, SCP4 and SCP6 groups were fed the CON diet during the starter phase (0‐14 d) and then the CON diet with respectively 2%, 4%, or 6% of SCPs from torula yeast (*Candida utilis*; provided by ARBIOM, Durham, NC, USA) as partial replacement for soybean during the grower and finisher phase (from day 15 to 42). All experimental diets were isoenergetic and with a similar amino acid profile, optimized to maintain the same ratio of total essential amino acids to total lysine (Table 1). According to data provided by the manufacturer (ARBIOM, Durham, NC, USA), the torula yeast (*Candida utilis*) derived SCPs used in this study contain approximately 55% crude protein, with an essential amino acid profile characterized by relatively high levels of lysine and leucine (both ~3.5%), moderate concentrations of threonine (3.0%), valine (2.5%), isoleucine (2.2%) and arginine (2.5%), and comparatively lower levels of sulphur‐containing amino acids, with methionine at ~0.6% and methionine plus cysteine at ~1.1%. In addition, these SCPs provide micronutrients, including minerals such as phosphorus (~1.5%), calcium (~0.3%), and zinc (~60 ppm), as well as vitamins, notably folic acid (< 40 ppm). To allow the inclusion of SCP while maintaining the same protein content among all experimental diets, the amount of soybean meal was reduced in SCP2, SCP4, and SCP6 of approximately 12, 23% and 35% in the grower phase, and 16, 31% and 47% in the finisher one, compared to CON.

**Table 1:** Diet composition in starter, grower, and finisher phases.

Feeding phase	Starter (0–14 d)	Grower (15–29 d)				Finisher (30–42 d)			
Group	CON	CON	SCP2	SCP4	SCP6	CON	SCP2	SCP4	SCP6
Ingredient (%)									
Corn	41.8	45.1	45.9	46.6	47.4	55.5	56.2	57.1	57.8
Wheat	15.0	15.0	15.0	15.0	15.0	10.0	10.0	10.0	10.0
Pea	3.00	3.00	3.00	3.00	3.00	3.00	3.00	3.00	3.00
Soybean toasted	0.00	5.00	5.00	5.00	5.00	5.00	5.00	5.00	5.00
Soybean meal	23.8	22.1	19.5	16.9	14.4	16.2	13.7	11.1	8.6
Corn gluten	3.00	0.00	0.00	0.00	0.00	0.00	0.00	0.00	0.00
Sunflower meal	3.00	3.00	3.00	3.00	3.00	3.00	3.00	3.00	3.00
Soybean oil	2.91	3.32	3.08	2.83	2.59	4.07	3.83	3.58	3.35
Potato protein concentrate 75%	3.51	0.00	0.00	0.00	0.00	0.00	0.00	0.00	0.00
Calcium carbonate	0.26	0.28	0.34	0.40	0.46	0.50	0.56	0.62	0.68
Dicalcium phosphate	1.48	0.89	0.82	0.76	0.69	0.54	0.48	0.41	0.35
Sodium chloride	0.31	0.29	0.29	0.29	0.28	0.28	0.27	0.27	0.26
Sodium bicarbonate	0.05	0.05	0.05	0.05	0.05	0.06	0.07	0.08	0.09
Choline chloride	0.12	0.10	0.10	0.10	0.11	0.06	0.06	0.06	0.06
Lysine sulphate	0.54	0.59	0.59	0.59	0.59	0.58	0.58	0.58	0.59
l‐Threonine (98%)	0.09	0.15	0.15	0.14	0.14	0.15	0.14	0.14	0.13
DL‐Methionine (99%)	0.28	0.33	0.34	0.34	0.34	0.31	0.31	0.32	0.32
Phytase	0.05	0.05	0.05	0.05	0.05	0.05	0.05	0.05	0.05
NSP enzyme (0.05%)	0.05	0.05	0.05	0.05	0.05	0.05	0.05	0.05	0.05
AA mix (37% Arg, 31% Val, 11% Ile)	0.05	0.12	0.12	0.12	0.12	0.05	0.03	0.02	0.00
Vitamin‐mineral premix^1^	0.50	0.45	0.45	0.45	0.45	0.30	0.30	0.30	0.30
Mycotoxin binder	0.10	0.00	0.00	0.00	0.00	0.00	0.00	0.00	0.00
Emulsifier 0.1%	0.05	0.05	0.05	0.05	0.05	0.05	0.05	0.05	0.05
SCP‐ inactive yeast (Torula)	0.00	0.00	2.00	4.00	6.00	0.00	2.00	4.00	6.00
Chemical composition									
Dry Matter (%)	88.0	87.97	88.0	88.1	88.1	87.9	87.9	88.0	88.1
Crude protein (%)	22.5	19.5	19.5	19.5	19.5	17.0	17.0	17.0	17.0
Total lipid (%)	4.89	6.25	6.04	5.83	5.62	7.21	7.00	6.79	6.58
Crude fibre (%)	3.06	3.15	3.09	3.02	2.96	2.97	2.90	2.84	2.77
Threonine (total, %)	0.95	0.85	0.85	0.85	0.85	0.75	0.75	0.75	0.75
Lysine (total, %)	1.40	1.26	1.26	1.26	1.26	1.10	1.10	1.10	1.10
Met + Cys (total, %)	1.05	0.97	0.97	0.97	0.97	0.87	0.87	0.87	0.87
Calcium (total, %)	0.72	0.58	0.58	0.58	0.58	0.52	0.52	0.52	0.52
Phosphorous (total, %)	0.62	0.52	0.52	0.53	0.53	0.43	0.44	0.44	0.44
AME (kcal/kg)	3050	3125	3125	3125	3125	3250	3251	3251	3251

Abbreviations: AA, amino acids; AME, apparent metabolisable energy; CON, control; Cys, cysteine; Met, methionine; SCP, single cell proteins.

^1^ The premix provides the following per kg of feed: vitamin A (retinyl acetate), 12,500 IU; vitamin D3, 3500 IU; 25‐hydroxy vitamin D_3_, 37.5 mg; vitamin E, 150 mg; vitamin C, 150 mg; vitamin K_3_, 6.75 mg; Vitamin B3 (niacin), 75 mg; Vitamin B1, 4 mg; Vitamin B2, 9 mg; Vitamin B6, 5.5 mg; Vitamin B12, 0.05 mg; folic acid, 3.0 mg; biotin, 0.35 mg; thiamine, 4.0 mg; Mn, 100 mg; Zn, 102 mg; Fe, 30 mg; Cu, 15 mg; I, 2.0 mg; Se, 0.35 mg.

Performance traits were recorded and calculated on a pen basis at each diet change (14 and 29 d), at slaughter (42 d), and for the overall period. At each time point, birds were weighed per pen to determine body weight, and daily weight gain was calculated from the difference between consecutive measurements. Feed intake, expressed on a daily basis, was calculated per pen as the difference between the feed provided at the beginning of the feeding phase and the residuals at the end of it. Feed conversion ratio was calculated as feed intake divided by body weight gain for each period. Calculations were corrected for mortality when applicable. Plasma samples were collected at 21 and 42 days on 10 and 15 birds/group, respectively, and then subjected to metabolome analysis (^1^H‐Nuclear Magnetic Resonance). At the end of the trial, on day 42, birds were processed at a commercial slaughterhouse. At the processing plant, birds were subjected to electrical stunning (200–220 mA, 1,500 Hz; Reg. 2009/1099/EC), killed through an automated severing device, and allowed bleeding. Then, carcasses were scalded (approx. 50°C–52°C for 280 s), plucked, eviscerated, chilled at 4°C, and then automatically deboned. Breasts (*n* = 12/group) were collected for post‐mortem analyses of meat technological properties and proximate composition. As a trait affecting performance and welfare (Hashimoto et al. 2013), the incidence and severity of footpad dermatitis (FPD) were assessed on all birds (1 foot/bird). Severity was determined according to a three‐point evaluation scale (Ekstrand et al. 1998): score 0 = no lesions, score 1 = mild lesions (< 0.8 cm), and score 2 = severe lesions (> 0.8 cm).

All experimental procedures were conducted according to the current European and Italian legislation (Dir. 2007/43/EC; Reg. 2009/1099/EC; Dir. 2010/63/EU) and approved by the Ethical Committee of the Alma Mater Studiorum ‐ University of Bologna and by the Italian Minister of Health (n. 1145/2020).

### 2.2 Technological Properties and Proximate Composition of Breast Meat

Ultimate pH was recorded on the anterior region of each *Pectoralis major* muscle using a portable pH metre (HI‐98163, Hanna Instruments Ltd., UK). The instrument was calibrated prior to measurements with standard buffer solutions at pH 4.01 and 7.01.

Colour was measured (in triplicate) at the same anatomical location using a Chroma Meter CR‐400 (Konica Minolta Sensing Inc., Osaka, Japan) under standardized conditions (illuminant C; 2° standard observer) and expressed as lightness (L*), redness (a*), and yellowness (b*) (CIE‐ Commission Internationale de l’Éclairage. 1976).

Water‐holding capacity was determined by calculating drip and cooking losses. A standardized meat sub‐sample (8 × 4 × 3 cm; approximately 80 g) was excised from the antero‐ventral area of each breast. After initial weighing, samples were placed on plastic racks in plastic boxes and stored at 4°C± 1°C for 48 h. Then, samples were gently blotted, reweighed, and the drip loss calculated as the percentage of weight lost following refrigerated storage (Petracci and Baéza 2011). Samples were vacuum‐packaged and heat‐treated in a water bath at 75°C until an internal temperature of 72°C was achieved. Cooking loss was expressed as the percentage of weight loss after thermal processing. Texture was assessed through the Warner–Bratzler shear test. Parallelepiped samples (4 × 1 × 1 cm) were excised parallel to the muscle fibre orientation. Measurements were performed with a TA‐HDi Heavy Duty texture analyser (Stable Micro Systems Ltd., Godalming, Surrey, UK) equipped with a 5‐kg load cell and a Warner–Bratzler shear blade. The crosshead speed was set at 2 mm/s, and results were expressed in kilograms.

Proximate composition (moisture, crude protein, total fat, and ash) was determined according to AOAC (1990) procedures. Moisture and ash content were quantified by drying (105°C for 18 h) and incinerating (550°C for 8 h) minced meat samples. Crude protein was calculated from total nitrogen content determined by the Kjeldahl method (AOAC 1990). The total lipid amount was obtained via chloroform–methanol extraction as reported by Folch et al. (1957).

### 2.3 Plasma Metabolome Profile

Blood samples obtained through wing vein withdrawal were collected into lithium‐heparin vials and centrifuged to get plasma, which was conserved at −80°C for ^1^H‐NMR spectrometry. For this analysis, which was carried out as described in a previous study (Zampiga et al. 2021), a ^1^H‐NMR solution was produced with D_2_O, containing 10 mmol/L of 3‐(trimethylsilyl)‐propionic‐2,2,3,3‐d4 acid sodium salt (TSP) as a reference for NMR chemical shift and 2 mmol/L NaN3 to avoid microbial proliferation. Plasma samples were centrifuged (18,630 × *g* for 15 min at 4°C), and an aliquot of the supernatant (0.65 mL) was mixed with 0.1 mL of the ^1^H‐NMR solution and centrifuged again under the same conditions. An AVANCE™ III spectrometer (Bruker, Milan, Italy) equipped with the Topspin software (v.3.5) was utilized for spectra recording. Temperature and frequency conditions were 298 K and 600.13 MHz, respectively. A CPMG‐filter (400 echoes with a τ of 400 µs and a 180° pulse of 24 µs, for a total filter of 330 ms) was applied to suppress the signals from broad resonances originating from large molecules, whereas the water residual signal was suppressed through pre‐saturation by employing the pulse sequence CPMGPR1D. Each spectrum was collected by summing up 256 transients constituted by 32,000 data points encompassing a window of 7184 Hz, divided by 5 s of relaxation delay. Topspin v3.5 was used to phase‐adjust the spectra, which were subsequently exported to ASCII format through the script convbin2asc and imported into the R software environment employing in‐house developed scripts. Signals were assigned to specific molecules by comparing their position in the spectra, shape, and multiplicity with the Human Metabolome Database (Wishart et al. 2007) and Chenomx software libraries (Chenomx Inc., Edmonton, AB, Canada, v10), by relying on the routines made available by Chenomx software. TSP was considered as an internal standard for the quantification of molecule concentration, while probabilistic quotient normalization (Dieterle et al. 2006) was applied to compensate for potential differences in water content among samples. As in Brugaletta et al. (2023a, 2023b), the concentration of molecules was determined according to the area of one of their signals, computed by the GSD (global spectra deconvolution) algorithm of MestReNova software (v14.2.0–26256; Mestrelab research S.L., Santiago De Compostela, Spain). A limit of quantification (LOQ) of 5 was considered. Before, a baseline adjustment was performed applying the Whittaker Smoother procedure and a line broadening of 0.3.

### 2.4 Statistical Analysis

All data were analysed using IBM® SPSS® Statistics (Version 28.0.1.1−14). Statistical models followed the structure described by Steel et al. (1997). Data were tested for normal distribution using the Shapiro–Wilk test, whereas homoscedasticity was assessed by means of the Levene test. Growth performance data were analysed using one‐way ANOVA followed by Tukey's post‐hoc test, considering the diet as the experimental factor and replicates as the experimental unit. FPD data were analysed for severity using a three‐point scale (0 = none, 1 = mild, 2 = severe), with the sampled animal as the experimental unit. A 95% confidence interval and Pearson's chi‐squared test assessed significance. Data on plasma metabolome as well as breast meat technological properties and proximate composition were analysed using one‐way ANOVA followed by Tukey's post‐hoc test, considering the diet as the experimental factor. Linear and quadratic responses to SCP inclusion were also assessed. Significance level for all tests was considered at a *p*‐value of ≤ 0.05.

## 3 Results

### 3.1 Growth Performance

Data summarizing the growth performance of broilers in starter, grower, finisher phases, and the entire trial are shown in Table 2. One‐day‐old male chicks were randomly distributed and weighed equally across the four experimental groups (*p* = 0.926).

**Table 2:** Growth performance of broilers in starter, grower, finisher phases, and the entire trial.

	CON	SCP2	SCP4	SCP6	SEM	*p*‐value
Hatchling weight (g/bird)	41.6	41.5	41.4	41.6	0.79	0.926
Starter period (0–14 d)						
Body weight (g/bird)	434.5	420.8	429.9	430.1	22.46	0.586
Daily weight gain (g/bird/d)^1^	28.0	27.3	28.1	27. 9	1.59	0.737
Daily feed intake (g/bird/d)^1^	36.4	35.9	35.1	36.1	1.63	0.365
Feed intake (kg/bird)^1^	0.509	0.503	0.492	0.506	0.02	0.365
Feed conversion ratio^1^	1.302	1.313	1.255	1.298	0.05	0.094
Mortality (%)	0.56	1.11	3.89	1.67	0.11	0.164
Grower period (15–29 d)						
Body weight (g/bird)	1665	1639	1616	1590	67.06	0.102
Daily weight gain (g/bird/d)^1^	81.6^**a**^	80.3^**ab**^	78.4^**ab**^	77.0^**b**^	3.43	**0.026**
Daily feed intake (g/bird/d)^1^	115.7	115.0	115.8	114.1	3.76	0.700
Feed intake (kg/bird)^1^	1.736	1.724	1.737	1.711	0.06	0.700
Feed conversion ratio^1^	1.419	1.433	1.480	1.483	0.08	0.160
Mortality (%)	0.56	0.59	0.56	0.00	0.07	0.818
Finisher period (30–42 d)						
Body weight (g/bird)	3083	3058	2988	2942	125.67	0.071
Daily weight gain (g/bird/d)^1^	109.5	110.3	107.5	105.9	5.80	0.339
Daily feed intake (g/bird/d)^1^	191.1^**a**^	188.7^**ab**^	186.6^**ab**^	183.7^**b**^	5.78	**0.050**
Feed intake (kg/bird)^1^	2.484^**a**^	2.452^**ab**^	2.426^**ab**^	2.388^**b**^	0.08	**0.050**
Feed conversion ratio^1^	1.750	1.712	1.742	1.737	0.08	0.766
Mortality (%)	0.63	0.00	0.00	0.63	0.06	0.594
Entire trial (0–42 d)						
Body weight (g/bird)	3083	3058	2988	2942	125.67	0.071
Daily weight gain(g/bird/d)^1^	72.1	71.6	69.8	68.8	3.01	0.070
Daily feed intake (g/bird/d)^1^	109.1	108.0	106.9	106.3	3.17	0.235
Feed intake (kg/bird)^1^	4.729	4.679	4.655	4.604	0.12	0.165
Feed conversion ratio^1^	1.603	1.596	1.619	1.626	0.06	0.626
Mortality (%)	1.67	1.67	4.44	1.67	0.12	0.199

Abbreviations: CON, control; SCP, single cell proteins; SEM, standard error mean.

^1^ Data are presented as the mean of 10 replicate pens/group and corrected for mortality.

Means within a row with different superscripts denote significant difference between groups, as derived from a one‐way ANOVA analysis (*p* ≤ 0.05).

Comparable performance was exhibited in starter and grower phases, with the only exception of daily weight gain in the latter phase (81.6 vs. 80.3 vs. 78.4 vs. 77.0 g/bird/day, respectively for CON, SCP2, SCP4, and SCP6; *p* < 0.05; linear fit < 0.01). In the finisher phase, daily feed intake and feed intake were higher in the CON group than in SCP6 one, with SCP2 and SCP4 presenting intermediate values (2.484 vs. 2.452 vs. 2.426 vs. 2.388 kg/bird, and 191.1 vs. 188.7 vs. 186.6 vs. 183.7 g/bird/d, respectively for CON, SCP2, SCP4, and SCP6, respectively; *p* ≤ 0.05; linear fit ≤ 0.01). In the overall trial period, a trend towards lower body weight and daily weight gain indicated a similar pattern according to the SCP inclusion percentage (3083 vs. 3058 vs. 2988 vs. 2942 g/bird, and 72.1 vs. 71.6 vs. 69.8 vs. 68.8 g/bird/day, respectively for CON, SCP2, SCP4, and SCP6; *p* = 0.071; linear fit < 0.01). However, no significant effect of the dietary treatments was observed for feed intake, feed conversion ratio, and mortality.

The incidence and severity of FPD yielded comparable results among groups (*p* = 0.519). Almost all birds had no lesions (score of 0; 98% vs. 94% vs. 95% vs. 98% respectively for CON, SCP2, SCP4, and SCP6), and only limited numbers had mild lesions (score of 1; 1% vs. 5% vs. 4% vs. 1% respectively for CON, SCP2, SCP4, and SCP6), or severe lesions (score of 2; 1% for all groups).

### 3.2 Meat Quality Traits and Proximate Composition

Data concerning the quality traits of breast meat and its proximate composition are reported in Table 3. Ultimate pH, drip loss, cooking loss, and shear force were similar across groups, with no significant differences observed. The same was found for lightness (L*) and redness (a*). On the other hand, a linear increase in yellowness (b*) was observed, increasing the inclusion level of SCPs in the feed formulation (7.67 vs. 8.57 vs. 9.11 vs. 10.38, respectively for CON, SCP2, SCP4, and SCP6; *p* < 0.001; linear fit < 0.01). As for the proximate composition, comparable values were observed across groups with no significant changes in the percentage of moisture, protein, fat, and ash.

**Table 3:** Meat quality and proximate chemical composition in breast filets of 42‐day‐old broilers.

	CON	SCP2	SCP4	SCP6	SEM	*p*‐value
Meat quality trait						
Ultimate pH	5.81	5.86	5.87	5.85	0.02	0.629
Lightness‐ L*	55.83	54.59	55.02	53.86	0.34	0.219
Redness‐ a*	2.65	2.64	2.58	2.09	0.17	0.600
Yellowness‐ b*	7.67^**c**^	8.57^**bc**^	9.11^**ab**^	10.38^**a**^	0.22	**< 0.001**
Drip loss (%)	3.24	2.75	2.24	2.89	0.19	0.719
Cooking loss (%)	22.9	22.0	22.0	23.6	0.58	0.799
Shear force (kg)	1.49	1.49	1.39	1.37	0.05	0.219
Proximate composition						
Moisture (%)	76.13	76.23	75.94	76.27	0.17	0.910
Crude protein (%)	22.14	21.53	21.79	21.89	0.12	0.340
Fat (%)	1.83	1.75	1.85	1.73	0.06	0.850
Ash (%)	1.33	1.30	1.30	1.22	0.03	0.630

Abbreviations: CON, control; SCP, single cell proteins; SEM, standard error mean.

Means within a row with different superscript letters denote significant differences among the groups, as derived from a one‐way ANOVA analysis and a Tukey‐HSD post‐hoc test (*p* ≤ 0.05). *N* = 12/group.

### 3.3 Plasma Metabolome

**Figure 1:**
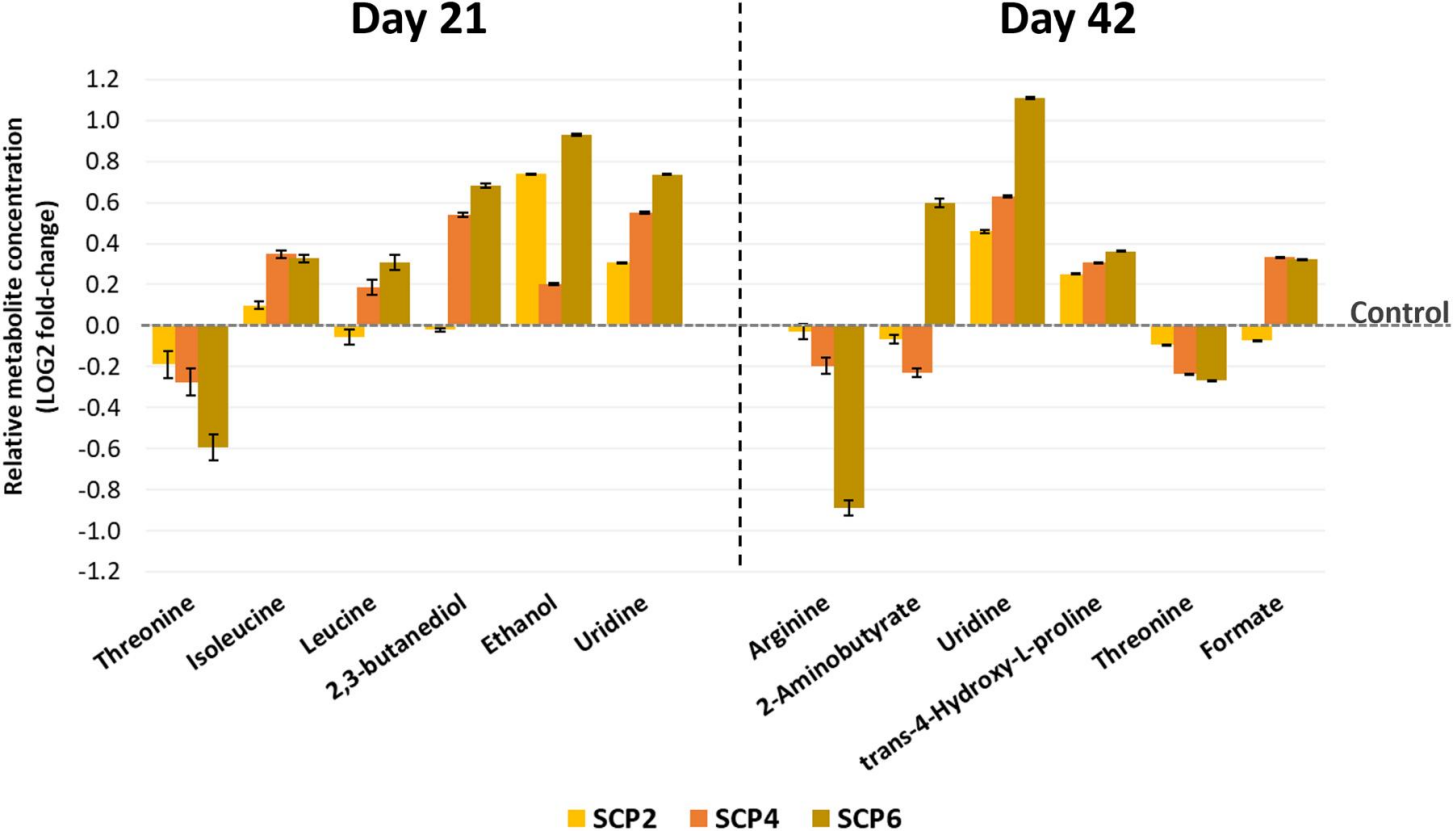
Relative metabolite concentration in significantly differing metabolites from SCP2, SCP4, and SCP6 groups, presented as LOG2 fold‐change versus CON. The LOG2 scale differences, were chosen as it provides a symmetric and intuitive representation of up‐ and down‐regulation, where positive and negative values reflect proportional increases and decreases, without affecting statistical significance, which was determined from analyses conducted on the original data. Differences between groups on days 21 and 42 were assessed by a one‐way ANOVA (*p* ≤ 0.05) and are presented for the non‐transformed data in Tables 4 and 5. *N* = 9 birds/group/day. [Color figure can be viewed at wileyonlinelibrary.com]

The concentration of plasma metabolites was analysed by ^1^H‐NMR, and a total of 54 metabolites were identified, of which 13 and 8 showed significant differences on days 21 and 42, respectively (Tables 4 and 5). The remaining metabolites that did not show significant differences are listed in Supplementary Tables 1 and 2. Fold‐changes were expressed as LOG2‐transformed values relative to CON. Figure 1 presents the LOG2 scale differences, chosen as it provides a symmetric and intuitive representation of up‐ and down‐regulation without affecting statistical significance, which was determined from analyses conducted on the original data.

**Table 4:** Concentration (mmol/L) of plasma metabolites of 21‐day‐old broilers.

Metabolite	CON	SCP2	SCP4	SCP6	SEM	*p*‐value
Threonine	2.06^**a**^	1.81^**a**^	1.70^**ab**^	1.36^**b**^	0.06	< 0.001
Betaine	1.37^**b**^	1.76^**a**^	1.40^**b**^	1.44^**b**^	0.05	< 0.001
Glycine	0.93^**b**^	1.15^**a**^	0.92^**b**^	1.01^**ab**^	0.03	< 0.001
Isoleucine	0.14^**b**^	0.15^**ab**^	0.18^**a**^	0.17^**a**^	0.005	< 0.001
Leucine	0.26^**b**^	0.25^**b**^	0.29^**ab**^	0.32^**a**^	0.01	0.011
Glycerol	0.38^**ab**^	0.26^**b**^	0.30^**ab**^	0.55^**a**^	0.04	0.020
2,3‐butanediol	0.10^**c**^	0.10^**c**^	0.15^**ab**^	0.16^**a**^	0.01	0.029
2‐Hydroxybutyrate	0.13^**b**^	0.41^**a**^	0.27^**ab**^	0.33^**ab**^	0.03	0.030
Asparagine	0.57^**a**^	0.53^**ab**^	0.43^**b**^	0.47^**ab**^	0.02	0.031
Ethanol	0.03^**b**^	0.05^**ab**^	0.04^**ab**^	0.06^**a**^	0.004	0.032
Formate	0.12^**b**^	0.15^**ab**^	0.16^**a**^	0.15^**ab**^	0.01	0.041
Uridine	0.03^**b**^	0.03^**ab**^	0.04^**ab**^	0.05^**a**^	0.003	0.048
Ascorbate	0.05^ab^	0.07^a^	0.07^a^	0.06^a^	0.002	0.049

^1^ H‐NMR analysis identified 54 plasma metabolites, of which 13 showed significant differences and are presented in the table; the remaining metabolites are listed in Supplementary Table 1. Means within a row with different superscripts denote significant difference between groups, as derived from a one‐way ANOVA analysis (*p* ≤ 0.05). *N* = 9/group.

**Table 5:** Concentration (mmol/L) of plasma metabolites of 42‐day‐old broilers.

Metabolite	CON	SCP2	SCP4	SCP6	SEM	*p*‐value
Arginine	0.45^**a**^	0.44^**a**^	0.39^**a**^	0.24^**b**^	0.02	< 0.001
2‐Aminobutyrate	0.05^**b**^	0.04^**b**^	0.04^**b**^	0.07^**a**^	0.003	< 0.001
Uridine	0.02^**c**^	0.03^**bc**^	0.03^**b**^	0.05^**a**^	0.002	< 0.001
Arabinose	0.11^**b**^	0.11^**b**^	0.12^**a**^	0.12^**ab**^	0.002	0.003
trans‐4‐Hydroxy‐L‐proline	0.19^**b**^	0.22^**ab**^	0.23^**ab**^	0.24^**a**^	0.01	0.034
Threonine	1.56^**a**^	1.46^**ab**^	1.32^**b**^	1.29^**b**^	0.04	0.050
Formate	0.18^**b**^	0.17^**b**^	0.22^**a**^	0.22^**a**^	0.01	0.050
Glucose	21.42^ab^	22.56^ab^	23.34^a^	23.17^a^	0.27	0.052

^1^ H‐NMR analysis identified 54 plasma metabolites, of which 8 showed significant differences and are presented in the table; the remaining metabolites are listed in Supplementary Table 2. Means within a row with different superscripts denote significant difference between groups, as derived from a one‐way ANOVA analysis (*p* ≤ 0.05). *N* = 9/group.

Focusing on differences attributable to SCPs inclusion levels, on day 21, Threonine concentrations were significantly lower in SCP6 compared to CON (*p* < 0.001), with a LOG2 fold‐change of −0.59. This value corresponds to a fold‐change of 2^−0.59^ = 0.66, indicating that Threonine levels in SCP6 were approximately 66% of those in CON, which represents a 34% decrease relative to CON. Opposingly, levels of Isoleucine (fold‐change 0.33; 26% increase), Ethanol (fold‐change 0.93; 91% increase), Uridine (fold‐change 0.74; 67% increase), were significantly higher in SCP6 compared to CON (*p* < 0.05), and levels of Leucine (fold‐change 0.31; 24% increase), 2,3‐butanediol (fold‐change 0.68; 60% increase), significantly higher compared to CON and also SCP2 (*p* < 0.03).

On day 42, levels of Arginine (fold‐change −0.89; 46% decrease) were significantly lower in SCP6 compared to CON and also SCP2, SCP4 (*p* < 0.001). Threonine levels (fold‐change −0.27; 17% decrease) were significantly lower in SCP6 and SCP4 compared to CON (*p* = 0.05). However, levels of 2‐Aminobutyrate (fold‐change 0.60; 51% increase), and Uridine (fold‐change 1.11; 116% increase) were significantly higher in SCP6 compared to CON and also SCP2, SCP4 (*p* < 0.001). The levels of trans‐4‐Hydroxy‐L‐proline (fold‐change 0.36; 29% increase) were significantly higher in SCP6 compared to CON (*p* = 0.034), and the levels of Formate (fold‐change 0.32; 25% increase) were significantly higher in SCP6 and SCP4 compared to CON and SCP2 (*p* = 0.05).

## 4 Discussion

This study evaluated the inclusion of torula yeast‐derived SCPs as a partial replacement of soybean meal in broiler diets. Overall, the results support the feasibility of incorporating SCPs at low levels from 15 to 42 days as indicated by production traits, breast meat quality and composition, and the plasma metabolome profile. The comparable growth performance across the entire trial indicates no substantial effect of soybean meal replacement by SCPs, yet the observed linear trend in body weight and daily weight gain for the entire trial, and the significant reductions in grower daily weight gain and finisher feed intake in the SCP6 group, suggest an upper limit where inclusion of 6% SCP begins to reduce growth performance. Our results differ from those reported in other monogastric terrestrial and aquatic species, where no adverse effects of torula SCPs inclusion were observed in weaning pigs (Espinosa et al. 2020) or sea bream (Busti et al. 2025). In fact, although diets were formulated to be isoenergetic and to maintain a similar amino acid profile, equivalent formulation may not necessarily ensure identical nutrient availability at the animal level when ingredients with potentially different nutrient digestibility are provided. Changes in the carbon source for torula yeast growth, the industrial processing method, and the dietary fat content were demonstrated to affect amino acid bioavailability in an in vitro porcine digestion model, which could help explain differences in growth performance (Verstrepen et al. 2023). Another potential limiting factor for the availability of certain amino acids is the cell wall structures present in yeast, which may reduce digestibility unless effectively degraded through processing or enzymatic strategies (Ritala et al. 2017). As for footpad dermatitis, the low incidence rate across groups and the absence of significant differences among them indicate an overall positive litter‐related outcome under the conditions of the present trial, an important indicator of welfare and the overall performance of broilers (Hashimoto et al. 2013).

As expected, breast meat quality traits were not affected by dietary treatment, with the exception of a progressive increase in yellowness as the level of SCP inclusion increased. Similarly, the proximate composition of breast muscle remained unchanged across groups, indicating the feasibility of partial inclusion of SCPs up to 6% without any impairment of meat quality. To date, no studies have specifically evaluated the effects of SCP supplementation on meat quality parameters in chickens; consequently, the present findings cannot be directly compared with prior reports. Although the underlying cause of yellowness increase was not directly investigated, this response may be associated with yeast‐derived components, such as ergosterol, present in *Candida utilis*. Ergosterol is biosynthetically related to carotenoids, which are known to affect meat pigmentation, through the common prenyl lipid precursor farnesyl diphosphate (FPP). Although carotenoids are not naturally synthesized by *Candida utilis*, it possesses precursors of carotenoids, as demonstrated by studies showing that introduction of the carotenogenic genes *crtE*, *crtB*, and *crtI* enables the biosynthesis of lycopene from FPP (Shimada et al. 1998). Another probable factor is that, as a compound, ergosterol exhibits yellowish coloration under exposure to light and air (PubChem CID: 444679). It is worth noting that the yellowing effect associated with SCP inclusion may represent a potential advantage, as consumer demand for yellow‐skinned chickens is increasing alongside the growing market for free‐range poultry (Baéza et al. 2022) and also in light of the limits imposed by the European legislation regarding the maximum dosage of synthetic yellow pigments in poultry diets (Regulation 2020/1400).

As for plasma metabolome analysis, overall limited differences were observed, as out of a total of 54 identified metabolites, 13 and 8 showed significant differences on days 21 and 42, respectively, and only six (for each examined day) were SCP inclusion dependent, supporting the overall feasibility of Torula yeast‐derived SCP supplementation. Nevertheless, a deeper look into changes in the metabolomic profile may enable a better understanding of physiological responses, even when no marked phenotypic changes are observed (Brugaletta et al. 2023a, 2023b). The lower threonine concentrations detected may reflect reduced threonine availability and correspond with a suggested upper limit for growth performance at SCP inclusion levels above 6%. As an essential amino acid, central to avian mucin synthesis and intestinal function and integrity (Fang et al. 1993; Kidd and Kerr 1996), changes in plasma threonine concentrations are unlikely to be the result of endogenous biosynthesis (Macelline et al. 2021). Higher plasma threonine concentrations have been associated with improved growth performance in broilers (Selle et al. 2016). The lower arginine levels observed in the SCP6 group at day 42 may further support a limitation at higher SCP inclusion levels. Arginine is an essential amino acid for chickens, supporting growth, muscle development, and intestinal function. Various studies have reported improvements in broiler growth performance and physiological function mediated by arginine (Basoo et al. 2012; Zampiga et al. 2018; Liu et al. 2019; Brugaletta et al. 2023a, 2023b). While the above‐mentioned metabolites showed lower values than CON, most significantly different metabolites showed higher values than CON. The observed higher plasma levels of leucine and isoleucine may reflect branched‐chain amino acid imbalance rather than enhanced anabolic signalling (Wu 2009). As performance traits were slightly reduced, it is plausible that imbalances between leucine, isoleucine, and valine disrupted effective amino acid utilization and subsequent growth performance by increasing the metabolic demand for the other branched‐chain amino acids or the first‐limiting amino acid (Harper et al. 1984; Wiltafsky et al. 2010; Selle et al. 2016). SCP inclusion also altered other metabolite levels, including uridine, a uracil nucleoside that is an essential component of RNA synthesis and plays an important role in glycogen synthesis (Yamamoto et al. 2011); trans‐4‐hydroxy‐L‐proline, which is an abundant constituent of collagen and elastin (Hu et al. 2022); Formate, which is involved in the provision of one‐carbon groups for several critical functions, including purine and thymidylate synthesis and methyl group provision (Bailey and Gregory 1999; Stover 2004; Brosnan et al. 2020); 2‐aminobutyrate, which is related to glutathione consumption under oxidative stress and activation of the glutathione synthetic pathway, accompanied by the production of ophthalmic acid from 2‐aminobutyric acid (Irino et al. 2016); and ethanol and 2,3‐butanediol are linked to acetyl‐CoA metabolism (Montgomery et al. 1993). These changes suggest that SCP inclusion may have influenced metabolic pathways related to energy metabolism, redox balance, and tissue turnover, reflecting a less favourable physiological status and further helping to explain the reduction in growth performance observed in the SCP6 group.

## 5 Conclusion

Together, the combined performance, welfare‐related, meat quality, and metabolomic data indicate that Torula yeast–derived SCPs can partially replace soybean meal in broiler diets at a dosage of 6% was associated with a tendency towards a reduction of final body weight and daily weight gain in the overall trial period and modified the plasma concentration of metabolites involved in important physiological functions, suggesting a possible upper limit for inclusion under the present formulation and feeding conditions of this study.

## Author Contributions

**Jonathan Dayan:** investigation, formal analysis, visualization, writing – original draft, writing – review and editing. **Marco Zampiga** and **Francesca Soglia:** investigation, formal analysis, visualization, writing – review and editing. **Elisa Benini:** visualization, writing – review and editing. **Luca Laghi**, **Massimiliano Petracci**, and **Federico Sirri:** resources, supervision, writing – review and editing.

## Ethics Statement

All experimental procedures were conducted according to the current European and Italian legislation (Dir. 2007/43/EC; Reg. 2009/1099/EC; Dir. 2010/63/EU) and approved by the Ethical Committee of the Alma Mater Studiorum ‐ University of Bologna and by the Italian Minister of Health (n. 1145/2020).

## Conflicts of Interest

The authors declare that the research was conducted in the absence of any commercial or financial relationships that could be construed as a potential conflict of interest.

## Supporting information


**Table S1:** Concentration (mmol/L) of plasma metabolites of 21‐day‐old broilers.**Table S2:** Concentration (mmol/L) of plasma metabolites of 42‐day‐old broilers.

## Data Availability

The data that support the findings of this study are available from the corresponding author upon reasonable request. All data supporting the findings of this study are included in the article/Supporting Materials; further inquiries can be directed to the corresponding author.
