# Supplementary material for: Evaluation of Torula Yeast (Candida utilis) Single Cell Proteins as a Partial Replacement for Soybean Meal in Broiler Diets: Effects on Productive Performance, Meat Quality Traits, and Plasma Metabolome
Source: J Anim Physiol Anim Nutr (Berl). 2026 Jun 1;110(5):674–84. doi: 10.1111/jpn.70082 (PMC13576590; doi:10.1111/jpn.70082)
Supplement: Supplementary file 1 [file JPN-110-674-s001.docx]

**Supplementary Table 1.** Concentration (mmol/L) of plasma metabolites of 21-day-old broilers

| **Metabolite concentration (mmol/L)** | **CON** | **SCP2** | **SCP4** | **SCP6** | **SEM** | **P-value** |
| --- | --- | --- | --- | --- | --- | --- |
| Dimethylamine | 0.03 | 0.03 | 0.02 | 0.01 | 0.003 | 0.084 |
| Arginine | 0.53 | 0.39 | 0.37 | 0.40 | 0.03 | 0.124 |
| myo-Inositol | 0.73 | 0.84 | 0.82 | 0.95 | 0.03 | 0.140 |
| Arabinose | 0.10 | 0.11 | 0.11 | 0.11 | 0.003 | 0.155 |
| Succinate | 0.12 | 0.15 | 0.14 | 0.12 | 0.01 | 0.170 |
| Dimethyl-Sulfone | 0.55 | 0.58 | 0.39 | 0.35 | 0.04 | 0.174 |
| Acetone | 0.02 | 0.02 | 0.02 | 0.02 | 0.001 | 0.184 |
| Glutamine | 2.31 | 2.15 | 2.18 | 2.50 | 0.06 | 0.192 |
| Lysine | 0.16 | 0.11 | 0.22 | 0.20 | 0.02 | 0.198 |
| 3-Hydroxyisobutyrate | 0.05 | 0.05 | 0.05 | 0.04 | 0.003 | 0.229 |
| 2-Aminobutyrate | 0.05 | 0.05 | 0.06 | 0.04 | 0.003 | 0.237 |
| Fumarate | 0.02 | 0.02 | 0.02 | 0.02 | 0.001 | 0.246 |
| Choline | 0.09 | 0.09 | 0.08 | 0.09 | 0.002 | 0.260 |
| Mannose | 0.06 | 0.07 | 0.09 | 0.07 | 0.01 | 0.279 |
| Citrate | 0.65 | 0.62 | 0.60 | 0.60 | 0.01 | 0.297 |
| Serine | 1.48 | 1.62 | 1.34 | 1.50 | 0.05 | 0.328 |
| Proline | 0.58 | 0.60 | 0.55 | 0.73 | 0.04 | 0.345 |
| Lactate | 15.91 | 18.09 | 16.66 | 14.96 | 0.64 | 0.371 |
| 3-Hydroxybutyrate | 0.15 | 0.14 | 0.19 | 0.16 | 0.01 | 0.379 |
| Histidine | 0.06 | 0.07 | 0.06 | 0.06 | 0.002 | 0.392 |
| TMAO | 0.04 | 0.05 | 0.05 | 0.04 | 0.002 | 0.415 |
| Beta-Alanine | 5.92 | 6.53 | 6.23 | 6.16 | 0.13 | 0.417 |
| Methanol | 0.11 | 0.12 | 0.13 | 0.09 | 0.01 | 0.420 |
| Pyruvate | 0.20 | 0.18 | 0.22 | 0.17 | 0.01 | 0.437 |
| trans-4-Hydroxy-L-proline | 0.20 | 0.19 | 0.21 | 0.21 | 0.01 | 0.443 |
| Creatine | 0.06 | 0.08 | 0.19 | 0.05 | 0.03 | 0.449 |
| N,N-Dimethylglycine | 0.20 | 0.22 | 0.20 | 0.21 | 0.004 | 0.449 |
| Methylmalonate | 0.15 | 0.11 | 0.14 | 0.22 | 0.02 | 0.450 |
| Methionine | 0.40 | 0.33 | 0.34 | 0.34 | 0.02 | 0.456 |
| Citramalate | 0.21 | 0.23 | 0.21 | 0.24 | 0.01 | 0.474 |
| Alanine | 2.18 | 2.06 | 1.96 | 2.24 | 0.07 | 0.501 |
| Sarcosine | 0.07 | 0.07 | 0.06 | 0.07 | 0.003 | 0.510 |
| Phenylalanine | 0.24 | 0.23 | 0.25 | 0.23 | 0.004 | 0.533 |
| Tyrosine | 0.30 | 0.28 | 0.30 | 0.27 | 0.01 | 0.602 |
| Carnosine | 0.06 | 0.06 | 0.06 | 0.07 | 0.002 | 0.638 |
| Aspartate | 0.19 | 0.22 | 0.21 | 0.22 | 0.01 | 0.643 |
| Acetate | 0.04 | 0.03 | 0.05 | 0.04 | 0.004 | 0.674 |
| Glucose | 20.31 | 21.48 | 21.78 | 20.96 | 0.45 | 0.687 |
| Isopropanol | 0.01 | 0.01 | 0.01 | 0.01 | 0.001 | 0.695 |
| Valine | 0.33 | 0.33 | 0.35 | 0.33 | 0.01 | 0.771 |
| Theophylline | 0.01 | 0.01 | 0.01 | 0.01 | 0.001 | 0.944 |

Concentrations of plasma metabolites showing non-significant differences as identified by ^1^H-NMR analysis. Means within a row with different superscripts denote significant difference between groups, as derived from a one-way ANOVA analysis (P ≤ 0.05). N= 9/group.

**Supplementary Table 2.** Concentration (mmol/L) of plasma metabolites of 42-day-old broilers

| **Metabolite concentration (mmol/L)** | **CON** | **SCP2** | **SCP4** | **SCP6** | **SEM** | **P-value** |
| --- | --- | --- | --- | --- | --- | --- |
| Lysine | 0.15 | 0.21 | 0.18 | 0.15 | 0.01 | 0.065 |
| Glycerol | 0.41 | 0.34 | 0.33 | 0.28 | 0.02 | 0.075 |
| Ethanol | 0.20 | 0.07 | 0.06 | 0.06 | 0.02 | 0.093 |
| Citrate | 0.69 | 0.75 | 0.79 | 0.72 | 0.01 | 0.094 |
| Aspartate | 0.28 | 0.33 | 0.31 | 0.24 | 0.01 | 0.104 |
| Glycine | 1.15 | 1.10 | 0.97 | 1.12 | 0.03 | 0.106 |
| N,N-Dimethylglycine | 0.15 | 0.16 | 0.17 | 0.18 | 0.004 | 0.108 |
| Serine | 1.57 | 1.52 | 1.47 | 1.37 | 0.03 | 0.116 |
| Creatine | 0.12 | 0.10 | 0.11 | 0.08 | 0.01 | 0.137 |
| Histidine | 0.08 | 0.07 | 0.08 | 0.06 | 0.003 | 0.145 |
| Carnosine | 0.06 | 0.07 | 0.06 | 0.05 | 0.003 | 0.184 |
| Citramalate | 0.20 | 0.19 | 0.21 | 0.17 | 0.01 | 0.190 |
| Pyruvate | 0.16 | 0.20 | 0.20 | 0.19 | 0.01 | 0.208 |
| Alanine | 2.19 | 2.32 | 2.29 | 2.48 | 0.05 | 0.258 |
| 3-Hydroxybutyrate | 0.14 | 0.18 | 0.28 | 0.20 | 0.02 | 0.265 |
| Acetone | 0.03 | 0.03 | 0.03 | 0.03 | 0.001 | 0.279 |
| Tyrosine | 0.40 | 0.34 | 0.36 | 0.34 | 0.01 | 0.282 |
| Acetate | 0.03 | 0.04 | 0.04 | 0.05 | 0.003 | 0.292 |
| TMAO | 0.04 | 0.05 | 0.06 | 0.06 | 0.005 | 0.339 |
| Succinate | 0.18 | 0.16 | 0.16 | 0.19 | 0.01 | 0.347 |
| 2-Hydroxybutyrate | 0.27 | 0.21 | 0.28 | 0.22 | 0.02 | 0.347 |
| Sarcosine | 0.05 | 0.09 | 0.06 | 0.05 | 0.01 | 0.367 |
| Methionine | 0.26 | 0.25 | 0.23 | 0.27 | 0.01 | 0.377 |
| Glutamine | 2.74 | 2.91 | 3.01 | 3.02 | 0.07 | 0.438 |
| Ascorbate | 0.07 | 0.07 | 0.07 | 0.08 | 0.003 | 0.474 |
| Methanol | 0.12 | 0.12 | 0.11 | 0.10 | 0.004 | 0.477 |
| Asparagine | 0.36 | 0.32 | 0.38 | 0.39 | 0.02 | 0.485 |
| Leucine | 0.32 | 0.29 | 0.32 | 0.31 | 0.01 | 0.519 |
| Valine | 0.37 | 0.36 | 0.35 | 0.35 | 0.01 | 0.533 |
| Lactate | 24.47 | 23.41 | 23.00 | 24.99 | 0.54 | 0.548 |
| Methylmalonate | 0.23 | 0.32 | 0.25 | 0.19 | 0.03 | 0.570 |
| 2,3-butanediol | 0.09 | 0.09 | 0.10 | 0.09 | 0.004 | 0.586 |
| Phenylalanine | 0.22 | 0.23 | 0.22 | 0.22 | 0.003 | 0.614 |
| Theophylline | 0.01 | 0.01 | 0.01 | 0.01 | 0.000 | 0.616 |
| Dimethylamine | 0.01 | 0.01 | 0.01 | 0.01 | 0.001 | 0.668 |
| Mannose | 0.07 | 0.07 | 0.07 | 0.07 | 0.002 | 0.687 |
| Proline | 0.61 | 0.59 | 0.64 | 0.66 | 0.02 | 0.697 |
| myo-Inositol | 1.07 | 1.16 | 1.14 | 1.14 | 0.03 | 0.705 |
| Betaine | 1.03 | 1.09 | 1.10 | 1.09 | 0.03 | 0.797 |
| Isoleucine | 0.16 | 0.17 | 0.16 | 0.17 | 0.003 | 0.865 |
| Fumarate | 0.02 | 0.02 | 0.02 | 0.02 | 0.001 | 0.919 |
| Choline | 0.07 | 0.07 | 0.07 | 0.07 | 0.002 | 0.930 |
| 3-Hydroxyisobutyrate | 0.03 | 0.03 | 0.03 | 0.03 | 0.001 | 0.965 |
| Dimethyl-Sulfone | 0.22 | 0.22 | 0.20 | 0.20 | 0.01 | 0.967 |
| Isopropanol | 0.01 | 0.01 | 0.01 | 0.01 | 0.0005 | 0.990 |
| Beta-Alanine | 5.95 | 5.92 | 5.91 | 5.91 | 0.07 | 0.996 |

Concentrations of plasma metabolites showing significant differences as identified by ^1^H-NMR analysis. Means within a row with different superscripts denote significant difference between groups, as derived from a one-way ANOVA analysis (P ≤ 0.05). N= 9/group.
